# Non-arteritic anterior ischemic optic neuropathy with Cilioretinal artery occlusion: a case report

**DOI:** 10.1186/s12886-019-1243-6

**Published:** 2019-11-14

**Authors:** Ya-Yun Yang, Ming-Shan He

**Affiliations:** 1 Department of Ophthalmology, Buddhist Tzu Chi General Hospital, Hualien, Taiwan; 2 Department of Ophthalmology and Visual Science, Tzu Chi University, Hualien, Taiwan

**Keywords:** Non-arteritic anterior ischemic optic neuropathy, Cilioretinal artery occlusion, Branch retinal artery occlusion

## Abstract

**Background:**

To describe a peculiar case of concurrent non-arteritic anterior ischemic optic neuropathy (NAION) and cilioretinal arteries occlusion (CLRAO) without other causative agents which responded well to intravenous and intravitreal injection of corticosteroids.

**Case presentation:**

A 41-year-old woman presented with painless vision loss in the right eye for 1 week. Fundus examinations showed marked disc swelling, flame-shaped hemorrhage over the superior nerve fiber area, and well-demarcated retinal ischemia superior to the fovea in the right eye. Under the impression of NAION with branch retinal artery occlusion, the patient was treated with intravenous and intravitreal injection of corticosteroids. Two months later, as the disc swelling and retinal ischemia resolved, we found that the occluded artery was the cilioretinal artery and not the ordinary branch retinal artery.

**Conclusions:**

CLRAO may be concomitant with the setting of NAION, the physicians should be aware that CLRAO may be misinterpreted as BRAO owing to profound disc edema during the early stages of the disease.

## Background

Non-arteritic anterior ischemic optic neuropathy (NAION) is due to acute ischemia of the optic nerve head (ONH), whose main supply of blood is from the circulation of the posterior ciliary arteries (PCA). The vast majority of NAION cases result from transient non-perfusion or hypoperfusion of ONH circulation [1]. Cilioretinal arteries also arise from short PCA. Thus, if retinal vascular occlusion occurs, the presence of a cilioretinal artery can significantly influence visual morbidity. It is interesting that although both the optic nerve head and cilioretinal arteries are supplied by PCA, concomitant anterior ischemic optic neuropathy (AION) and cilioretinal arteries occlusion (CLRAO) are uncommon in clinical practice. If it does occur, it is almost always arteritic and always pathognomonic for giant cell arteritis; other causes have been reported, including overdose of Viagra® [2]. Here we report on a peculiar case involving concurrent NAION and CLRAO without other causative agents.

## Case presentation

**Fig. 1:**
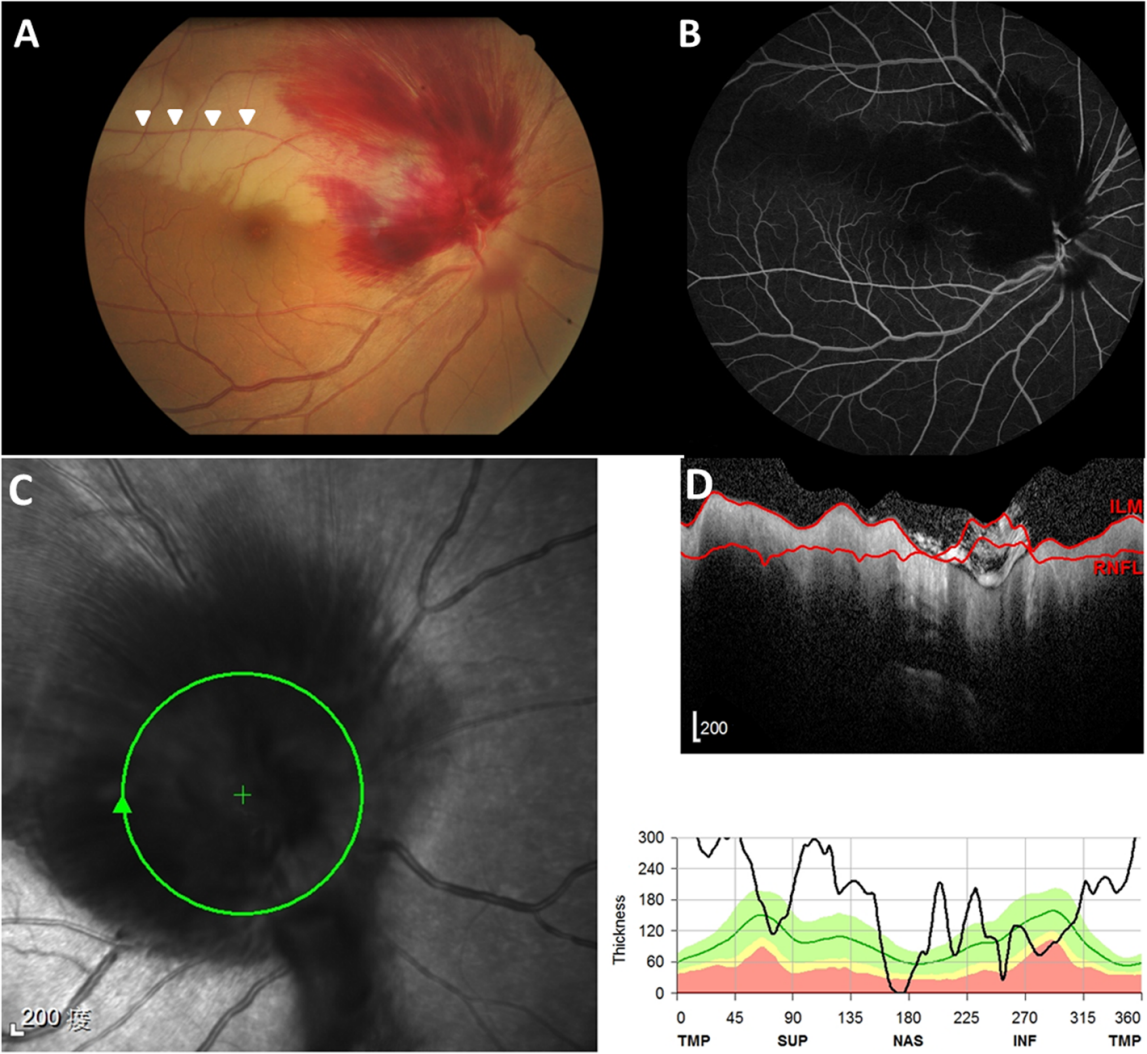
**a**. The fundus showed marked disc swelling, flame-shaped hemorrhaging over the superior nerve fiber area and well-demarcated retinal ischemia along with branch retinal artery (arrowheads) superior to the fovea in the right eye. **b**. Fluorescein angiography disclosed a filling defect of retinal arterial circulation superior to the fovea correlated with retinal ischemia. **c**. Infrared image and **d**. Corresponding OCT retinal nerve fiber layer (RNFL) scan revealed profound disc swelling over the superior and temporal nerve fiber area of the right eye

**Fig. 2:**
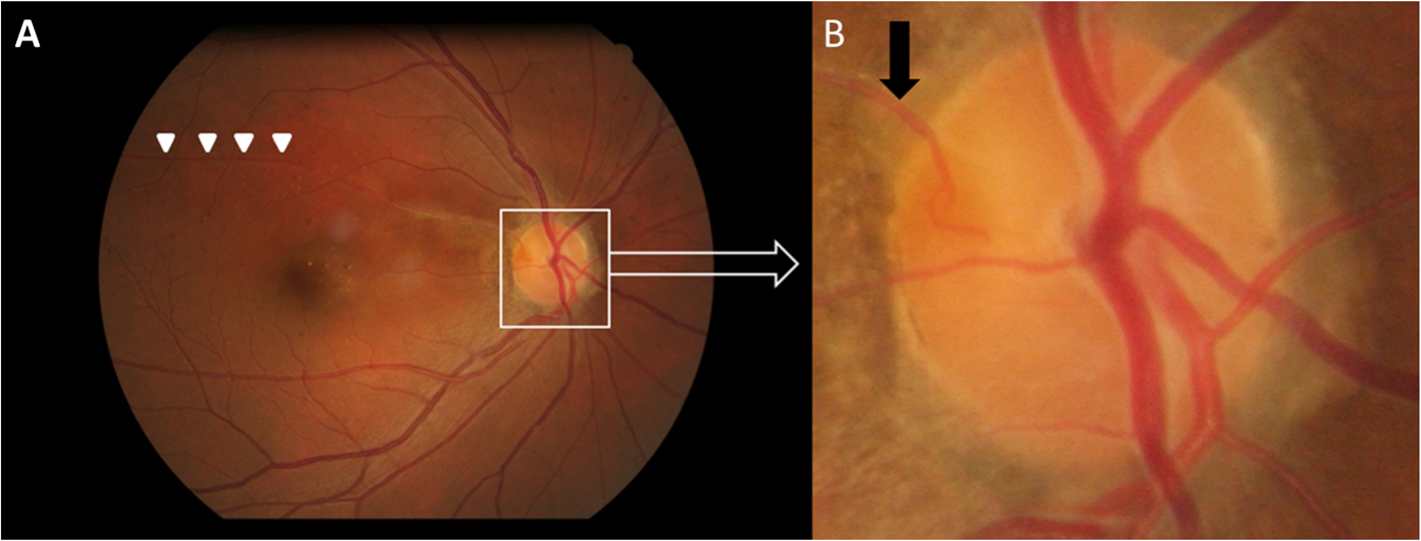
**a** Six months later, the fundus showed resolved disc swelling and retinal ischemia along with branch retinal artery (arrowheads). **b**. A high magnification image of the right disc disclosed the occluded artery was found to be the cilioretinal artery (black arrow)

A 41-year-old woman with a history of hypertension visited our hospital due to sudden onset of painless vision loss in the right eye for 1 week. Her height is 5′; body weight is 49 Kg with a body mass index is 22 kg/m2. She doesn’t have sleep apnea, and the vision loss occurred while she woke up. Visual acuity was 20/200 OD and 20/40 OS. Fundus and OCT (Optical Coherence Tomography) examinations showed marked disc swelling, flame-shaped hemorrhaging over the superior and temporal nerve fiber area (Fig. 1a, c, d), and well-demarcated retinal ischemia superior to the fovea in the right eye (Fig. 1a), with an absent optic cup appearance of the left eye. In addition, we found a relative afferent pupillary defect in the right eye. Visual field examination showed peripheral constriction and inferior arcuate defect of the right eye and normal of the left eye. Fluorescein angiography disclosed a filling defect of retinal arterial circulation superior to the fovea correlated with retinal ischemia and blocked fluorescence due to profound retinal hemorrhaging over the disc in the right eye (Fig. 1b). Examination revealed blood pressure was 158/105 mmHg. Cardiac and carotid doppler sonography were normal. Laboratory examinations for the complete blood count, antinuclear antibody, protein C/S, and homocysteine were within normal ranges; the erythrocyte sediment rate (ESR) was 6 mm/hour; total cholesterol was 234 mg/dL. Under the impression of NAION with branch retinal artery occlusion (BRAO), the patient was admitted for intravenous methylprednisolone pulse therapy for 3 days (total dose: 3000 mg) followed by gradual tapering oral prednisolone and one intravitreal injection of triamcinolone. Two months later, as the disc swelling and retinal ischemia resolved, we found that the occluded artery was the cilioretinal artery and not the ordinary branch retinal artery (Fig. 2). Visual acuity improved to 20/25 in the right eye 6 months after the treatment. Disc revealed a pale change in the superior and temporal part with an absent optic cup.

## Discussion and conclusion

The cilioretinal artery is present in 20% of the population, and the PCA supplies the optic nerve head as well as the cilioretinal artery. When CLRAO is combined with AION, it is nearly pathognomonic for giant cell arteritis associated arteritic-AION [3]. Our patient was relatively young, had normal ESR and did not have temporal tenderness or headache, which made giant cell arteritis unlikely. Furthermore, unlike small peripapillary hemorrhaging in most NAION cases, profound hemorrhaging along superior fiber area revealed poorer local circulation compared with others. In our case, marked optic disc swelling inherent to NAION may have resulted in local compartmental effects around the disc, which may have further compromised the circulation of the cilioretinal artery. This then led to concomitant CLRAO. Furthermore, the physicians should be aware that CLRAO may be misinterpreted as BRAO owing to profound disc edema during the early stages of the disease.

Little is known regarding the optimal management of NAION. The main debate in recent decades is whether or not to use steroids [4]. Although there is no consensus, steroids are thought to decrease optic disc edema and improve circulation in the optic nerve head [5]. The visual loss in NAION was investigated to be due not just to ischemia but also to associated inflammation, and this has shown in both animals with experimental NAION and in the few cases of acute NAION in humans that have been studied [6]. Some case series also showed treatment efficacy in intravitreal injection of triamcinolone for high intraocular steroid concentration without systemic adverse effects [7]. Our case showed that CLRAO may be concomitant with the setting of NAION. High dose intravenous corticosteroids and intravitreal injection of triamcinolone may decrease optic disc edema and improve circulation in both the optic nerve head and cilioretinal artery with favorable results.

In conclusion, CLRAO may be concomitant with the setting of NAION, the physicians should be aware that CLRAO may be misinterpreted as BRAO owing to profound disc edema during the early stages of the disease.

## Data Availability

All the data supporting the conclusions of this article is included in the present article.

## Abbreviations

AION: Anterior ischemic optic neuropathy
BRAO: Branch retinal artery occlusion
CLRAO: Cilioretinal arteries occlusion
NAION: Non-arteritic anterior ischemic optic neuropathy
OCT: Optical Coherence Tomography
ONH: Optic nerve head
PCA: Posterior ciliary arteries
